# Vitamin B6 deficient plants display increased sensitivity to high light and photo-oxidative stress

**DOI:** 10.1186/1471-2229-9-130

**Published:** 2009-11-10

**Authors:** Michel Havaux, Brigitte Ksas, Agnieszka Szewczyk, Dominique Rumeau, Fabrice Franck, Stefano Caffarri, Christian Triantaphylidès

**Affiliations:** 1 Commissariat à l'Energie Atomique (CEA), Institut de Biologie Environnementale et de Biotechnologie, Laboratoire d'Ecophysiologie Moléculaire des Plantes, 13108 Saint-Paul-lez-Durance, France; 2 Centre National de la Recherche Scientifique (CNRS), Unité Mixte de Recherche Biologie Végétale et Microbiologie Environnementales, 13108 Saint-Paul-lez-Durance, France; 3 Université Aix-Marseille, 13108 Saint-Paul-lez-Durance, France; 4 Pharmaceutical Faculty of the Collegium Medicum, Jagiellonian University, Krakow, Poland; 5 Laboratory of Plant Biochemistry and Photobiology, Institute of Plant Biology, University of Liège, 4000-Liège, Belgium

## Abstract

**Background:**

Vitamin B6 is a collective term for a group of six interconvertible compounds: pyridoxine, pyridoxal, pyridoxamine and their phosphorylated derivatives. Vitamin B6 plays essential roles as a cofactor in a range of biochemical reactions. In addition, vitamin B6 is able to quench reactive oxygen species *in vitro*, and exogenously applied vitamin B6 protects plant cells against cell death induced by singlet oxygen (^1^O_2_). These results raise the important question as to whether plants employ vitamin B6 as an antioxidant to protect themselves against reactive oxygen species.

**Results:**

The *pdx1.3 *mutation affects the vitamin B6 biosynthesis enzyme, pyridoxal synthase (PDX1), and leads to a reduction of the vitamin B6 concentration in *Arabidopsis thaliana *leaves. Although leaves of the *pdx1.3 Arabidopsis *mutant contained less chlorophyll than wild-type leaves, we found that vitamin B6 deficiency did not significantly impact photosynthetic performance or shoot and root growth. Chlorophyll loss was associated with an increase in the chlorophyll *a*/*b *ratio and a selective decrease in the abundance of several PSII antenna proteins (Lhcb1/2, Lhcb6). These changes were strongly dependent on light intensity, with high light amplifying the difference between *pdx1.3 *and the wild type. When leaf discs were exposed to exogenous ^1^O_2_, lipid peroxidation in *pdx1.3 *was increased relative to the wild type; this effect was not observed with superoxide or hydrogen peroxide. When leaf discs or whole plants were exposed to excess light energy, ^1^O_2_-mediated lipid peroxidation was enhanced in leaves of the *pdx1.3 *mutant relative to the wild type. High light also caused an increased level of ^1^O_2 _in vitamin B6-deficient leaves. Combining the *pdx1.3 *mutation with mutations affecting the level of 'classical' quenchers of ^1^O_2 _(zeaxanthin, tocopherols) resulted in a highly photosensitive phenotype.

**Conclusion:**

This study demonstrates that vitamin B6 has a function in the *in vivo *antioxidant defense of plants. Thus, the antioxidant activity of vitamin B6 inferred from *in vitro *studies is confirmed *in planta*. Together with the finding that chloroplasts contain vitamin B6 compounds, the data show that vitamin B6 functions as a photoprotector that limits ^1^O_2 _accumulation in high light and prevents ^1^O_2_-mediated oxidative damage.

## Background

Natural vitamin B6 consists of six interconvertible compounds, pyridoxine, pyridoxal, pyridoxamine and their phosphorylated derivatives, pyridoxine 5'-phosphate, pyridoxal 5'-phosphate and pyridoxamine 5'-phosphate [1-3]. Most bacteria, fungi and plants possess vitamin B6 biosynthesis pathways, but mammals must acquire the vitamin in their diet. In plants, the *de novo *pathway of vitamin B6 biosynthesis relies on two proteins, PDX1 and PDX2, which function as a glutamine amidotransferase and produce pyridoxal-phosphate from intermediates of glycolysis and the pentose phosphate pathway [4,5]. PDX1 and PDX2 work together, with the latter protein as the glutaminase and the former as the synthase domain.

Vitamin B6 plays essential roles as a cofactor in a wide range of biochemical reactions, predominantly in amino acid metabolism [6,7]. Recently, besides their classical role as coenzymes, a new function has emerged for the various vitamin B6 compounds in cellular antioxidant defense. A link between vitamin B6 and oxidative stress was originally established in the phytopathogenic fungus *Cercospora nicotianae*. Mutant strains were identified that were particularly vulnerable to their own toxin cercosporin, a photosensitizer that produces singlet oxygen (^1^O_2_) in the light [8]. Unexpectedly, cloning of the mutant genes in *C. nicotianae *revealed that the mutated fungi were affected in a gene of the vitamin B6 biosynthesis pathway [9]. Subsequently, it was shown *in vitro *that vitamin B6 is able to quench ^1^O_2 _with a high efficiency [9,10]. Additional analyses revealed that vitamin B6 is also able to quench superoxide [11]. The antioxidant capacities of vitamin B6 were confirmed in yeast or animal cell cultures supplied with exogenous vitamin B6 compounds and exposed to different oxidative treatments [12-16]. Similarly, exogenously applied vitamin B6 was found to protect plant protoplasts against ^1^O_2_-induced cell death [17]. These *in vitro *results indicate that vitamin B6 is a potential antioxidant and raise the question as to whether plants employ vitamin B6 to protect themselves against reactive oxygen species (ROS), particularly ^1^O_2_. Several mutants of *Arabidopsis thaliana *defective in vitamin B6 biosynthesis have been recently isolated which could help answering this question. A knock out of the single *PDX2 *gene is lethal for *Arabidopsis *[4]. There are 3 homologues of *PDX1 *in *Arabidopsis*, *PDX1.1*, *PDX1.2 *and *PDX1.3*. Two of these (*PDX1.1 *and *PDX1.3*) have been shown to be functional in vitamin B6 synthesis [4]. While disruption of both genes causes lethality, the single mutants *pdx1.1 *and *pdx1.3 *are viable, indicating that one gene can compensate, at least partially, for the lack of the other. However, *PDX1.3 *is more highly expressed than *PDX1.1*, and a *PDX1.3 *knockout accumulates less vitamin B6 --about 30-40% of the wild type (WT) level) and has a more severe mutant phenotype in sterile medium [18-20]. Thus, *PDX1.3 *appears to be more important for vitamin B6 synthesis than *PDX1.1*.

When grown in sterile medium in the absence of vitamin B6, seedlings of the *pdx1.3 *mutant are strongly reduced in shoot growth and primary root growth [18,19,21,22]. Under these conditions, mutant seedlings were also found to be more sensitive to the ^1^O_2_-generating dye Rose Bengal, to salt stress and to UV radiation relative to WT seedlings [21]. Although this is consistent with the idea that vitamin B6 could play a role *in planta *as an antioxidant, it is difficult to draw a definite conclusion because of the rather severe phenotype of the mutant in sterile culture. Interestingly, when grown on soil, the mutant phenotype of the *pdx1.3 *mutant was much less pronounced. The reason for the less severe phenotype in soil is unknown. It has been suggested that there is a source of the vitamin in the soil [18]. However, the vitamin B6 concentration in the leaves of *pdx1.3 *mutant plants grown on soil remains very low compared to WT [19,20]. Alternatively, it is possible that growth in sterile medium in a Petri dish represents a form of stress to which plants with low levels of vitamin B6 are more sensitive. In this study, we took advantage of the nearly normal development of the vitamin B6-deficient *pdx1.3 Arabidopsis *mutant grown on soil to explore in detail the possibility that this vitamin functions as a photoprotector and an antioxidant in plants. We show that vitamin B6 acts as a new class of ^1^O_2 _quencher, thereby protecting plants against photooxidative stress.

## Results

### Growth and leaf chlorophyll content of *pdx1 *plants

**Figure 1:**
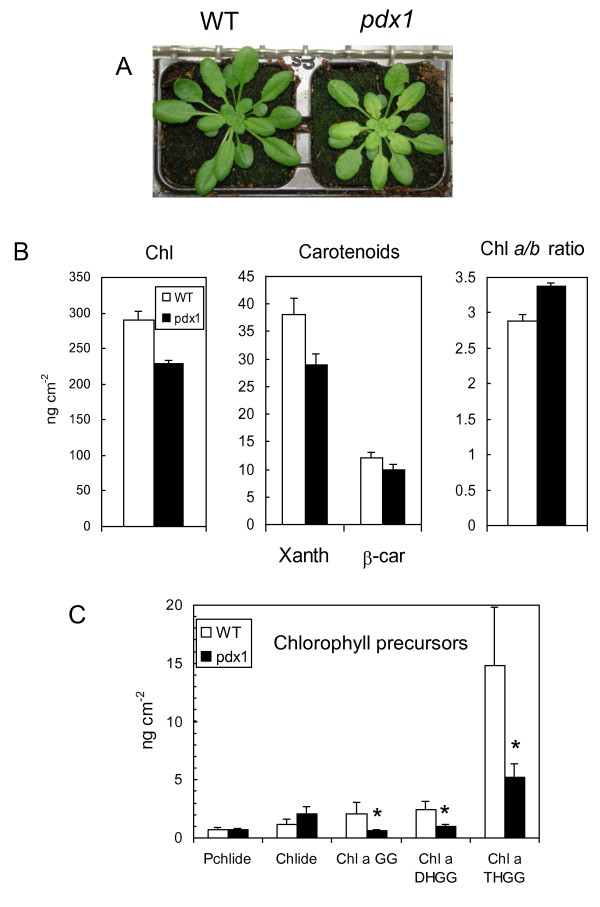
**Pigment content of young leaves of WT *Arabidopsis *and of the *pdx1 *mutant**. A) Plants aged 4 weeks. B) Chlorophyll and carotenoid content of young leaves. Chl, total chlorophyll; Xanth, xanthophylls; β-car, β-carotene. C) Level of various chlorophyll precursors in young leaves: Pchlide, protochlorophyllide; Chlide, chlorophyllide; GG-, DHGG- and THGG-Chl, geranylgeranyl-chlorophyll, dihydrogeranylgeranyl-chlorophyll and tetrahydrogeranylgeranyl-chlorophyll, respectively. Data are mean values of 4 measurements + SD. *, significantly different from the WT value with *P *< 0.01 (t test).

**Figure 2:**
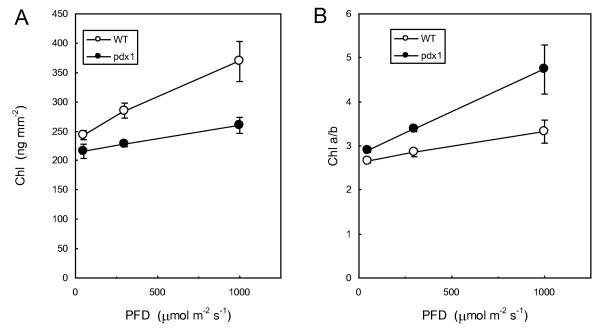
**A) Chlorophyll content and B) chlorophyll *a/b *ratio in leaves of WT and *pdx1 *plants grown at different PFDs**. Data are mean values of 3 measurements ± SD.

Vitamin B6-deficient *pdx1.3 *plants grown on soil (abbreviated as *pdx1 *hereafter) looked similar to WT plants, except that young leaves in the center of the rosette were paler (Fig. 1A) as previously reported [18,21]. This was due to a decrease in photosynthetic pigments (Fig. 1B): both chlorophylls (Chl) and carotenoids were reduced by about 15-20%, and this was accompanied by a significant increase in the Chl *a*/*b *ratio. This reduction of the pigment content tended to disappear in mature, well developed mutant leaves. We also measured the concentration of various Chl precursors in young leaves (Fig. 1C). No significant change was observed in protochlorophyllide (PChlide) and chlorophyllide (Chlide) levels between WT and mutant leaves. In contrast, a decrease in the geranylgeranylated forms of Chl, namely geranylgeranyl Chl (GG-Chl), dihydrogeranylgeranyl Chl (DHGG-Chl) and tetrahydrogeranylgeranyl Chl (THGG-Chl) was found in young leaves of the *pdx1 *mutant. It is known from studies of etiolated seedlings that GG-Chl is formed through a preferential esterification of Chlide by geranylgeranyl disphosphate catalyzed by the enzyme Chl synthase [23-25]. GG-Chl is then reduced stepwise to Chl via DHGG-Chl and THGG-Chl by geranylgeranyl reductase [26]. Therefore, the marked decrease in GG-Chl and other geranylgeranylated intermediates in leaves of the *pdx1 *mutant suggests that the Chl synthase activity is somehow affected by the *pdx1 *mutation, ultimately leading to a reduction in Chl concentration in the leaves. Therefore, it is likely that either the catalytic activity of Chl synthase itself is inhibited or that levels of the substrate geranylgeranyl diphosphate are more limiting. However, the unchanged level of tocopherols in the *pdx1 *mutant (see below) would suggest that levels of geranylgeranyl phosphate are not limiting. Moreover, a rice mutant with impaired Chlide esterification by Chl synthase has a phenotype that strongly resembles *pdx1 *mutants: decreased Chl levels were associated with an increased Chl *a*/*b *ratio in young plants, and these effects progressively disappeared as leaves matured [27]. We also found that the change in Chl content of leaves of the *pdx1 *mutant relative to WT leaves was strongly dependent on light intensity (Fig. 2): the difference in Chl concentration and in the Chl *a*/*b *ratio between WT and *pdx1 *was strongly attenuated when plants were grown in low light (80-100 μmol photons m^-2 ^s^-1^) and was enhanced when plants were grown in high light (1000 μmol m^-2 ^s^-1^).

**Figure 3:**
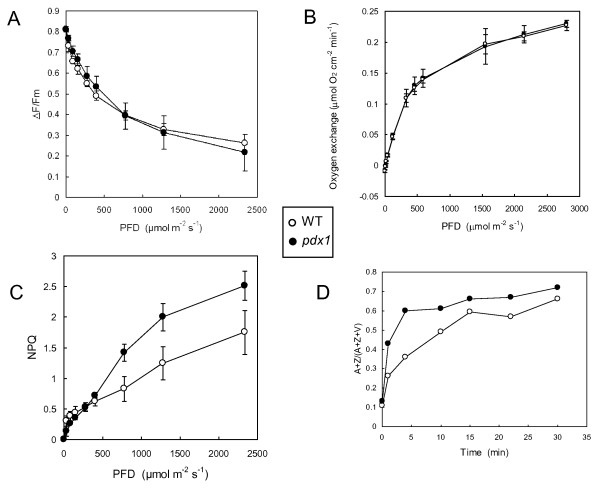
**Photosynthetic parameters of WT *Arabidopsis *leaves and leaves of the *pdx1 *mutant grown under control conditions (150-200 μmol m^-2 ^s^-1^, 25°C)**. A) Quantum yield of PSII photochemistry (ΔF/Fm'), B) oxygen exchange and C) NPQ measured at different PFDs. Data are mean values of 3 or 4 measurements ± SD. D) Light-induced conversion of violaxanthin (V) into zeaxanthin (Z) and antheraxanthin (A), as calculated by the equation (A+Z)/(V+A+Z). Zeaxanthin synthesis was induced by white light of PFD 1000 μmol m^-2 ^s^-1^. Each point corresponds to a different leaf (1 measurement per point).

The decrease in photosynthetic pigments in leaves of the *pdx1 *mutant was not associated with substantial changes in photosynthetic electron transport. The quantum yield of linear electron transport measured by Chl fluorometry was comparable in WT and *pdx1 *leaves (Fig. 3A). Similarly, the rate of O_2 _evolution measured with a Clark electrode did not appear to be affected by the *pdx1 *mutation (Fig. 3B). Also, neither shoot growth or root growth were significantly affected by inactivation of the *PDX1.3 *gene (Additional File 1). Normal development of vitamin B6-deficient shoot grown on soil was previously reported [18,21]. Clearly this was also the case for root development in soil.

We observed a difference in nonphotochemical energy quenching (NPQ) between WT leaves and leaves of the *pdx1 *mutant, with NPQ being enhanced in the latter leaves, particularly at high photon flux densities (PFDs) above 500 μmol photons m^-2 ^s^-1 ^(Fig. 3C). NPQ is a photoprotective mechanism that requires a transthylakoid pH gradient and the synthesis of zeaxanthin from violaxanthin in the light-harvesting antennae of PSII [28,29]. The increased NPQ in the *pdx1 *mutant is thus consistent with the increased rate of photoconversion of violaxanthin to zeaxanthin: zeaxanthin synthesis in high light was faster, and the final extent of conversion was increased in the *pdx1 *mutant relative to WT (Fig. 3D).

### *In vitro *sensitivity of vitamin B6-deficient leaves to ROS

**Figure 4:**
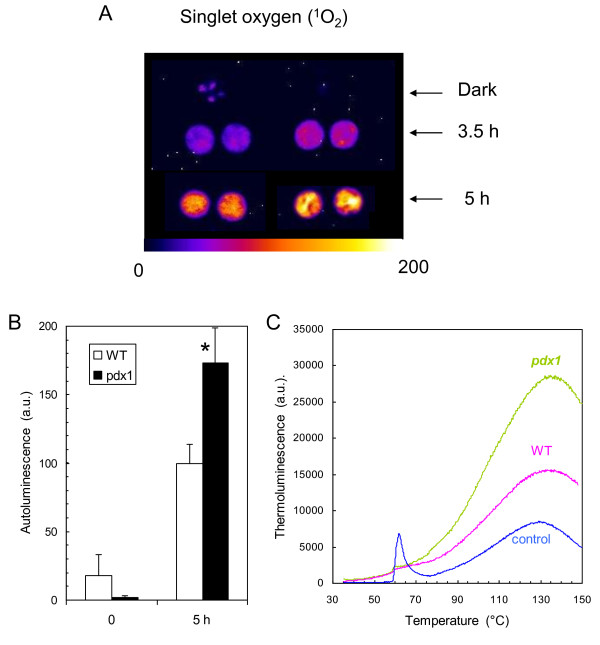
**Oxidative stress in *Arabidopsis *leaf discs (WT and *pdx1*) exposed to the ^1^O_2 _generator eosin (0.5%)**. A) Autoluminescence imaging of leaf discs exposed for 3.5 h or 5 h to eosin in the light (400 μmol photons m^-2 ^s^-1^). 'Dark' corresponds to eosin-infiltrated leaf discs kept in the dark for 5 h. B) Autoluminescence intensity in leaf discs exposed for 0 or 5 h to eosin in the light. Data are mean values of 10 measurements + SD. *, significantly different from the WT value with *P *< 0.001 (t test). C) Thermoluminescence band at high temperature (ca. 135°C) in leaf discs exposed for 5 h to eosin in the light. Control, leaf discs from *pdx1 *kept in eosin in the dark. Control WT disks (not shown) was in the same thermoluminescence intensity range. The band peaking at ca. 60°C in the control is typical of *Arabidopsis*. Its origin is unknown; it is not related to lipid peroxidation and could be due to thermolysis of a (yet unidentified) volatile compound [84].

Leaf discs were exposed to eosin, a xanthene dye that generates ^1^O_2 _in the light [30]. Illuminating leaf discs floating on a solution (0.5%) of eosin has been previously shown to cause leaf photooxidation and lipid peroxidation [30,31]. We visualized the effect of eosin by autoluminescence imaging. This technique measures the faint light emitted by triplet carbonyls and ^1^O_2_, the by-products of the slow and spontaneous decomposition of lipid hydroperoxides and endoperoxides [32-34]. Deactivation of excited carbonyls and ^1^O_2 _produces photons (in the blue and red spectral regions, respectively) which can be recorded with a high-sensitivity, cooled CCD (charge coupled device) camera [34]. This technique has been used to map lipid peroxidation and oxidative stress in various biological materials including detached leaves [35], whole plants [36,37], animals [38] and humans [39]. As shown in Fig. 4A, ^1^O_2_-induced lipid peroxidation was associated with a marked enhancement of leaf disc autoluminescence, as expected. Interestingly, the increase in autoluminescence was more pronounced in discs punched out from *pdx1 *leaves than in WT discs (Fig. 4A). We quantified the autoluminescence intensity, and we found a 50%-increase in the *pdx1 *mutant relative to WT (Fig. 4B). Thus, the *pdx1 *mutant appeared to be more sensitive to ^1^O_2 _toxicity than WT. This was confirmed by thermoluminescence analyses of lipid peroxidation (Fig. 4C). Thermal decomposition of lipid hydroperoxides is associated with photon emission in the 120-140°C range [33,40]. The amplitude of the thermoluminescence band peaking at ~135°C has been correlated in previous studies with the extent of lipid peroxidation as measured biochemically [33,36,41]. The 135°C band amplitude was noticeably higher in eosin treated leaf discs taken from *pdx1 *than from the WT. Using HPLC, we also found that the level of malondialdehyde, a 3-carbon aldehyde produced during lipid peroxidation, was 29% higher in *pdx1 *leaf discs than in WT discs after the eosin treatment (3 repetitions, data not shown). Together these results show that eosin treatment results in significantly increased lipid peroxidation in the mutant.

In contrast to ^1^O_2_, other ROS such as hydrogen peroxide and superoxide did not induce different amounts of photooxidation between mutant and WT leaf discs (Additional File 2). Although exposure of leaf discs to both ROS enhanced autoluminescence, this effect was similar in WT and *pdx1*. Similarly, the 135°C thermoluminescence band of *pdx1 *and WT leaf discs after H_2_O_2 _and superoxide treatment were indistinguishable (data not shown).

### Vitamin B6-deficient plants are more sensitive to ^1^O_2_-mediated lipid peroxidation than WT leaves

**Figure 5:**
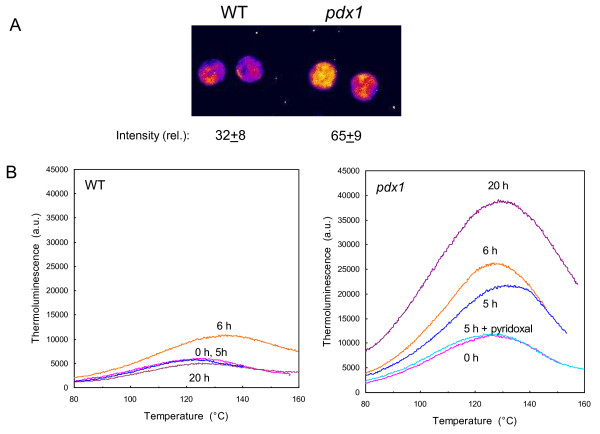
**Photooxidative stress in leaf discs (WT and *pdx1*)**. A) Autoluminescence of leaf discs exposed for 6 h to 1500 μmol m^-2 ^s^-1 ^at 10°C. B) Thermoluminescence band at high temperature (ca. 135°C) in leaf discs exposed to high light stress for 0, 5, 6 or 20 h. The thermoluminescence signal of discs taken from leaves of the *pdx1 *mutant and preinfiltrated with vitamin B6 (2 mM) is also shown (5 h + vitamin B6).

^1^O_2 _was recently shown to be the major ROS involved in photooxidative damage to leaves [42]. A combination of low temperature and high light is known to be particularly favorable for inducing photooxidative stress in higher-plant leaves [43]. Therefore, we exposed leaf discs to a high photon flux density (PFD) of 1000 μmol photons m^-2 ^s^-1 ^at low temperature (10°C). This treatment induced lipid peroxidation, as measured by autoluminescence (Fig. 5A) and thermoluminescence (Fig. 5B). Leaf discs from the *pdx1 *mutant were clearly more sensitive to the high light treatment than WT discs: both signals were enhanced in the mutant compared to WT. When leaf discs taken from the *pdx1 *mutant were infiltrated with vitamin B6 before the light treatment, the increased thermoluminescence relative to WT was lost, confirming that exogenous vitamin B6 can function as an antioxidant [17].

**Figure 6:**
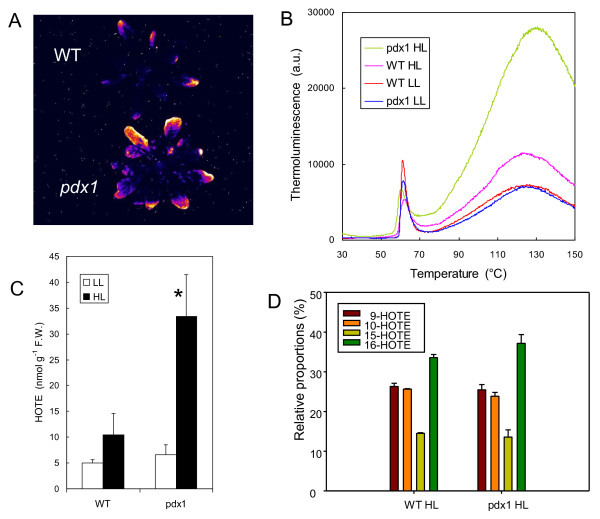
**Photooxidative stress of whole *Arabidopsis *plants (WT and *pdx1*)**. A) Autoluminescence imaging of lipid peroxidation after high light stress (2d, 6°C, 1500 μmol m^-2 ^s^-1^). B) Thermoluminescence signal of WT leaves and leaves of the *pdx1 *mutant before and after high light stress (LL and HL, respectively). C) Lipid hydroperoxide level (HOTE) in leaves of control and high light-stressed WT and *pdx1 *plants. *, significantly different from the WT value with *P *< 0.015 (t test). D) Distribution of lipid hydroperoxide (HOTE) isomers in leaves of control and high-light stressed WT and *pdx1 *plants. Data are mean values of 3 to 5 measurements + SD.

The high photosensitivity of vitamin B6-deficient leaf discs prompted us to investigate the responses of whole plants to photooxidative stress conditions. Figure 6 shows the effect of 2-d exposure of *Arabidopsis *plants to photooxidative stress induced by very high light (1500 μmol photons m^-2 ^s^-1^) at low temperature (6°C) on lipid peroxidation. Again, autoluminescence emission was much higher in *pdx1 *than in WT after this treatment (Fig. 6A). This was particularly visible in the external leaves, in agreement with previous studies that have emphasized the higher sensitivity of mature leaves to oxidative stress relative to young, developing leaves [*e.g*. [31,44]]. This observation indicates that the increased sensitivity of *pdx1 *to photooxidative stress is not directly attributable to the low-Chl phenotype of *pdx1 *which was visible mainly in the young leaves.

The differential sensitivity of the *pdx1 *mutant and WT to light stress was confirmed by thermoluminescence measurements (Fig. 6B) and also by HPLC analyses of lipid hydroperoxide concentrations (Fig. 6C). The level of HOTE (hydroxyl octadecatrienoic acid), the product of the oxidation of linolenic acid (the major fatty acid in plant leaves) doubled in WT plants after light stress. In *pdx1 *the HOTE concentration increased by a factor of 5. Figure 6D shows the relative proportions of the different HOTE isomers during lipid peroxidation induced by high light stress. Isomers specific to ^1^O_2 _(10-HOTE and 15-HOTE, [45]) were present in high amounts, and their level relative to the isomers 9-HOTE and 16-HOTE, which are produced by all ROS (free radicals and ^1^O_2_) was typical of ^1^O_2 _attack on polyunsatured fatty acids (see [42]). Thus, one can conclude that *pdx1 *plants are more sensitive to endogenous ^1^O_2 _production than WT plants.

### ^1^O_2 _levels during illumination are enhanced in the *pdx1 *mutant

**Figure 7:**
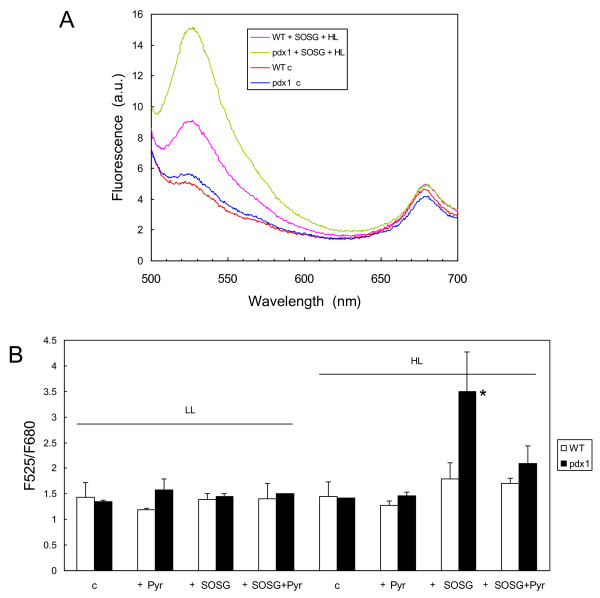
**Fluorescence of SOGS in WT and mutant (*pdx1*) leaves exposed to high light**. A) Fluorescence of leaves infiltrated with SOGS after exposure to white light (HL = 450 μmol photon m^-2 ^s^-1 ^for 40 min). Controls (= c) were kept in dim light before fluorescence measurements. B) Fluorescence ratio F525/F680 of WT leaves and mutant leaves infiltrated with SOGS and/or vitamin B6 before or after illumination. Data are mean values of 3 measurements + SD. *, significantly different from the WT value with *P *< 0.025 (t test).

Singlet oxygen sensor green (SOSG) reagent is a fluorescein derivative compound that is selective to ^1^O_2 _with no appreciable response to superoxide and hydroxyl radical [46]. In the presence of ^1^O_2_, it emits a green fluorescence that peaks at 525 nm. However, this fluorescent probe has a relatively low stability in the light, so that the use of this probe to measure ^1^O_2 _production should be restricted to short illumination only. Figure 7A shows the fluorescence spectrum of *Arabidopsis *leaves infiltrated under pressure with SOSG and illuminated for 40 min at a PFD of 400 μmol photons m^-2 ^s^-1^. SOSG fluorescence at 525 nm was well visible in the fluorescence emission spectrum of the illuminated leaves. This fluorescence was enhanced in *pdx1 *relative to WT, indicating an increased level of ^1^O_2 _in the former plants. Figure 7B shows the fluorescence emission at 525 nm (F525) normalized to the fluorescence of chlorophylls at 680 nm (F680) in leaves infiltrated with SOSG, with vitamin B6 or with both. The only condition that caused a significant increase in the F525/F680 ratio, indicative of an increased production of ^1^O_2_, was the illumination of SOSG-infiltrated leaves of the *pdx1 *mutant. Interestingly, the photoinduced increase in the F525/F680 ratio of *pdx1 *leaves was lost when leaves were infiltrated with vitamin B6 in addition to SOSG. This loss of SOSG fluorescence indicates that exogenous vitamin B6 can quench ^1^O_2 _*in vivo*, thus confirming *in vitro *data [10].

### The *pdx1 *mutation enhances the photosensitivity of the *vte1 npq1* mutant

**Figure 8:**
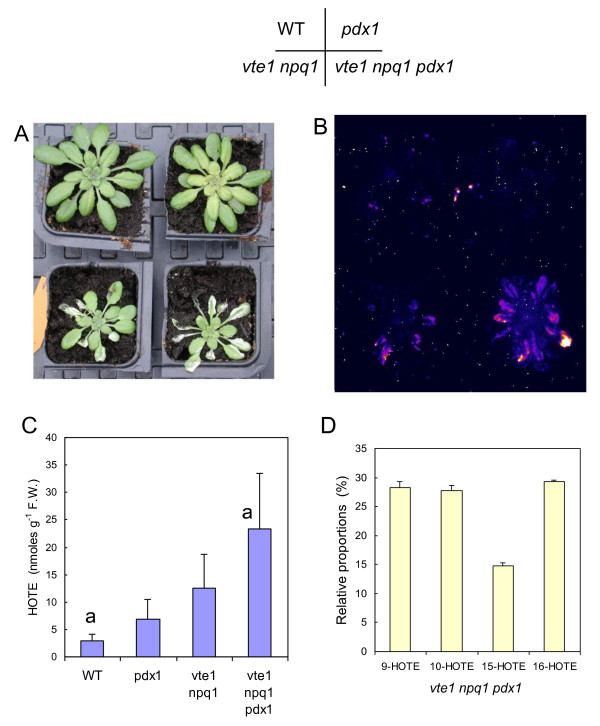
**Effects of high light stress (1000 μmol photons m^-2 ^s^-1 ^at 10°C for 2 d) on WT plants and on *pdx1*, *vte1 npq1 *and *vte1 npq1 pdx1 *mutant plants**. A) Plants after the high light treatment. B) Autoluminescence imaging of lipid peroxidation. C) HOTE level. a, significantly different with *P *< 0.03 (t test). D) Distribution of HOTE isomers in leaves of the *vte1 npq1 pdx1 *triple mutant exposed to the high light treatment. Data are mean values of 3 or 4 measurements + SD.

The *vte1 npq1 *double mutant is deficient in two major ^1^O_2 _quenchers, vitamin E (tocopherols) and the carotenoid zeaxanthin [47]. *Vte1 npq1 *is photosensitive, exhibiting oxidative stress and lipid peroxidation in high light [42,47]. This is illustrated in Fig. 8 where *vte1 npq1 *plants were exposed to a rather moderate light stress (white light of PFD 1000 μmol m^-2 ^s^-1 ^at 10°C). This treatment brought about leaf bleaching (Fig. 8A) and increased autoluminescence (Fig. 8B). On the contrary, both WT and *pdx1 *plants appeared to be resistant to this treatment. Similarly, the single mutants *vte1 *and *npq1 *did not display symptoms of photooxidative damage under these conditions (data not shown). The *vte1 npq1 *mutant was crossed with the *pdx1 *single mutant to generate a triple mutant (*vte1 npq1 pdx1*) deficient in vitamins E and B6 and in zeaxanthin. The triple mutant exhibited an extreme sensitivity to high light: most leaves bleached (Fig. 8A) and leaf autoluminescence increased markedly (Fig. 8B). We also measured the HOTE concentration in leaves (Fig. 8C), which was higher in the triple mutant than in the double or single mutants. Thus, removing vitamin B6 in the *vte1 npq1 *background led to a highly photosensitive phenotype. Analysis of the lipid peroxidation signature indicated that lipid peroxidation in the triple mutant was mediated by ^1^O_2 _(Fig. 8D). The high photosensitivity of leaves of the *vte1 npq1 pdx1 *triple mutant compared to leaves of the *vte1 npq1 *and *pdx1 *mutants suggests that there is some overlap in the functions of vitamin B6 and the zeaxanthin-vitamin E duo.

### Protective mechanisms against ^1^O_2 _in leaves of the *pdx1 *mutant

**Figure 9:**
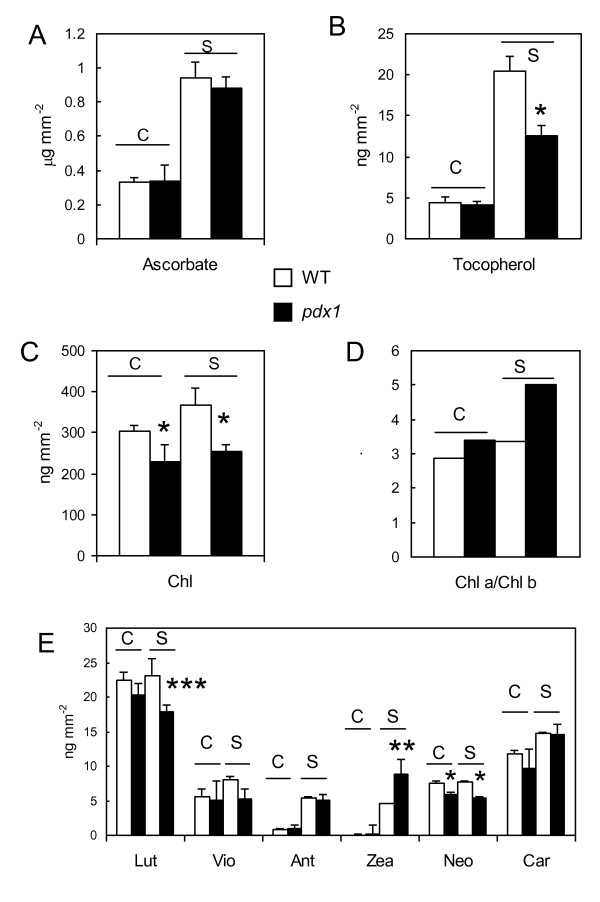
**Levels of chlorophyll and various antioxidants in WT leaves and leaves of *pdx1 *after long-term exposure to high light (1000 μmol m^-2 ^s^-1^, 10°C, 7d)**. A) Ascorbate, B) α-Tocopherol, C) Total chlorophyll, D) Chlorophyll *a*/*b *ratio, E) β-carotene (car) and xanthophylls (lutein (lut), violaxanthin (vio), antheraxanthin (ant), zeaxanthin (zea), neoxanthin (neo)). Data are mean values of 3 measurements + SD. C = control plants; S = plants exposed to the high light treatment. *, ** and ***, significantly different from the WT value with *P *< 0.001, 0.035 and 0.01, respectively (t test). White bars, WT; black bars, *pdx1 *mutant.

Figure 8 shows that *pdx1 *plants are able to tolerate high light, provided the stress is not too severe. We analyzed the level of various antioxidant compounds in *pdx1 *and WT plants during acclimation for 7 days to a PFD of 1000 μmol m^-2 ^s^-1^. Carotenoids and tocopherols are major quenchers of ^1^O_2 _in plant leaves while ascorbate is one of the most efficient scavengers of ^1^O_2 _[48]. Under control growth conditions, the ascorbate and tocopherol content of *pdx1 *and WT plants was similar. Light acclimation led to a comparable increase in ascorbate, in WT and *pdx1 *(Fig. 9A). Tocopherol was increased as well, but this change was less pronounced in *pdx1 *(Fig. 9B). This could be due to the consumption of tocopherol by increased oxidative stress in the mutant. Although the total Chl level (on a leaf area basis) did not change during photoacclimation (Fig. 9C), the Chl *a*/*b *ratio increased, especially in *pdx1 *(Fig. 9D). The most obvious change in carotenoid composition was an accumulation of antheraxanthin and zeaxanthin, which was more pronounced in the *pdx1 *mutant than in WT (Fig. 9E). β-Carotene also increased, but by a similar amount in *pdx1 *and WT. Lutein and neoxanthin did not change significantly during photoacclimation although they were slightly reduced in the mutant compared with WT. This reduction reflects a decrease in the PSII antenna size in the mutant (see below). The Chl-to-carotenoid ratio differed noticeably between WT and *pdx1*, falling from 4.17 to 3.89 and from 3.93 to 2.76 respectively during high light acclimation. Accumulation of carotenoids, especially zeaxanthin, and the putative consumption of α-tocopherol by oxidation suggests that the *pdx1 *mutant senses a higher level of photostress than WT.

### PSII antenna size is decreased in leaves of the *pdx1 *mutant

**Figure 10:**
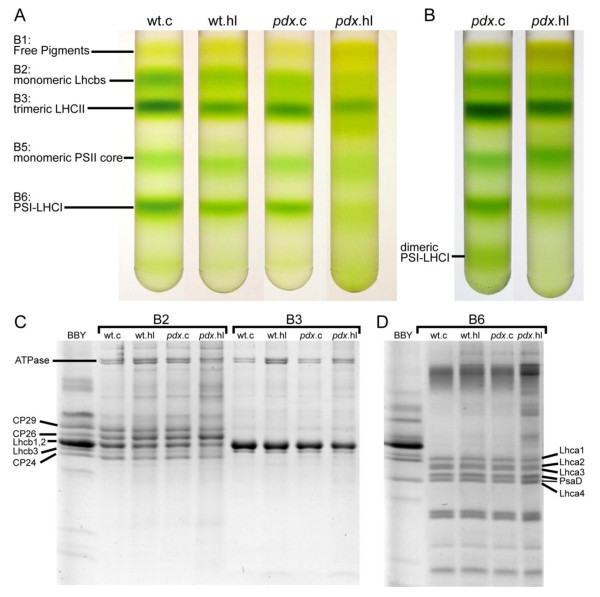
**A) Separation of pigmented photosynthetic complexes of thylakoids prepared from leaves of WT and *pdx1 *by solubilization in 0.8% dodecylmaltoside and ultracentrifugation on sucrose gradient**. Thylakoids were prepared from leaves of WT and *pdx1 *grown in low light (c, 200 μmol photons m^-2 ^s^-1^) or acclimated for 7 d to high light (hl, 1000 μmol m^-2 ^s^-1^). B1, free pigments; B2, monomeric Lhcb antennae; B3, LHCII trimers; B5, PSII core (monomeric), B6, PSI-LHCI supercomplex. The B4 band (LHCII-CP29-CP24 supercomplex, see [85]) is not visible in this gradient. B) Ultracentrifugation gradient of thylakoids (*pdx1*, c and hl) solubilized in 1.2% dodecylmaltoside. In the control *pdx1 *sample, an additional band appeared in the bottom of the gradient, which was hardly visible at 0.8% dodecylmaltoside and which corresponded to dimeric PSI-LHCI. This is presumably due to an artificial aggregation the high detergent concentration used in this preparation as previously found [86]; the same phenomenon was observed with WT thylakoids (data not shown). C and D) SDS-PAGE separation of the B2, B3 and B6 bands using two different buffer systems: tricine (C) and urea (D). See ref. [87] for identification of the bands. BBY = PSII-enriched membranes used as a reference for the PSII proteins.

The decreased Chl levels and increased Chl *a*/*b *ratio of *pxd1 *mutants (particularly at high PFD, Fig. 2) suggest that there is a differential adjustment of the photosynthetic complexes to the light environment in mutant compared to WT plants. Therefore, we analyzed the relative abundance of Chl-containing photosynthetic complexes in thylakoids prepared from WT and *pdx1*. The pigmented protein complexes of thylakoids were solubilized in 0.8% α-dodecylmaltoside and were separated by ultracentrifugation on sucrose gradient (Fig. 10A). As expected, acclimation of WT leaves to high light (1000 μmol m^-2 ^s^-1^) brought about a substantial decrease in the PSII antenna system (monomeric Lhcb and trimeric LHCII; B2 and B3 bands in Fig. 10A, respectively) relative to the PSII reaction center (B5 band). The PSI-LHCI supercomplex (B6 band) was also reduced during photoacclimation. Rather surprisingly the profile of thylakoids isolated from young leaves of low light-grown *pdx1 *plants was very similar to the profile of high light-grown WT plants. High light-grown *pdx1 *leaves showed a rather extreme situation: the PSII antennae were strongly reduced compared to the PSII core and the abundance of PSI-LHCI supercomplexes was extremely low. Long-term acclimation of *pdx1 *to high light was also associated with an increased level of free carotenoids (B1 band). Thus, the enhancement of the carotenoid/Chl ratio in leaves of the *pdx1 *mutant seems to be largely due to unbound carotenoids. However, the quality of the separation of the photosynthetic complexes of thylakoids prepared from high light-acclimated leaves of *pdx1 *was poor in 0.8% α-dodecylmaltoside, presumably because of a high lipid/protein ratio. Consequently, a higher α-dodecylmaltoside concentration (1.2%) was used to improve solubilization of thylakoids prepared from *pdx1 *leaves after acclimation to high light (Fig. 10B). By comparison with low light-grown *pdx1 *plants, the profile obtained with high-light treated *pdx1 *at this detergent concentration confirmed that the effects of high light were drastic in the mutant, with a strong decrease in PSI-LHCI and PSII antenna size and an increase in the level of free pigments (Fig. 10B).

A global reduction of the PSII antenna system (gradient fractions B2 and B3 vs. B5) could explain the increase in the Chl *a*/*b *ratio in the *pdx1 *mutant. However, the absorption spectra of the B2 and B3 bands showed that the light-harvesting complexes of PSII themselves contain less Chl *b *(Additional File 3), suggesting that the composition of these bands was modified. This prompted us to analyze the protein composition of the different bands by SDS-PAGE. Two different buffer systems were used: Tricine (Fig. 10C) and Laemmli-urea (Fig. 10D). The former system allows a good separation of the Lhcb polypeptides whereas the latter system is more appropriate for separating the Lhca proteins. In WT, acclimation to high light resulted in the decreased relative abundance of several PSII antennae (Lhcb1-2 and CP24, also named Lhcb6) and the increased relative abundance of CP26 (Lhcb5) with respect to control conditions. The abundance of CP29 (Lhcb4) was little affected (Fig. 10C). Low-light grown *pdx1 *plants showed similar changes in the relative abundances of Lhcb1-2, CP24 and CP26 indicating that even under low light this mutant suffers light stress comparable to that of the WT at a PFD of 1000 μmol m^-2 ^s^-1^. These changes were strongly amplified when *pdx1 *was exposed to high light. Since CP26 and CP29 have a higher Chl *a*/*b *ratio than other Lhcb antennae [49], the relative enhancement of these antennae might help contribute to the increased Chl *a*/*b *ratio in *pdx1*. The Chl *a*/*b *ratio of band B2 was particularly high (2.9) in *pdx1 *plants grown under high light. Band B2 consists of a mixture of different monomeric antennae that usually have Chl *a*/*b *ratios between 1.2 and 3.0 [49]. Therefore the high Chl *a*/*b *ratio of the B2 band *pdx1 *plants cannot simply be explained by a decrease in the abundance of the Chl *b*-rich monomers. Instead there must be an increased Chl *a*/*b *ratio within the Lhcb complex itself. This could be explained by either a reduced Chl *b *availability as a result of stress that results in Chl *a*-rich folding of the Lhc complexes, or else by the preferential accumulation of specific Lhcb isoforms that are rich in Chl *a*, as previously suggested for maize [50]. We also observed a higher abundance of ATPase relative to antenna proteins under high light (Fig. 10C and 10D). However a precise quantification is not possible from these gels since ATPase fragments into several subcomplexes during gradient centrifugation, with the most intact complex migrating in B6 together with PSI. However, we were able to further confirm the higher abundance of ATPase relative to Chl-binding complexes by SDS-PAGE separation of total thylakoid proteins (data not shown).

Changes in the relative proportions of the Lhca proteins in response to high light and/or in *pdx1 *were much less pronounced than those occurring in the PSII antenna system (Fig. 10D). Nevertheless, a relative increase in PsaD and possibly Lhca4 abundance seemed to occur in *pdx1 *plants that had been acclimatised to high light (Fig. 10D).

Together, the data of Figs. 9 and 10 suggest that vitamin B6-deficient leaves sensed a higher level of light stress at a given PFD and over-reacted to increasing PFD compared to WT leaves. Incidentally, the smaller antenna system of *pdx1 *was not associated with substantial changes in photosynthetic electron transport efficiency (Fig. 3). This is consistent with previous studies of PSII antenna mutants of Arabidopsis which have shown that rather strong reductions of the antenna system do not necessarily affect the photochemical activity of leaves [e.g. [51]].

### Vitamin B6 accumulation during high light acclimation

**Figure 11:**
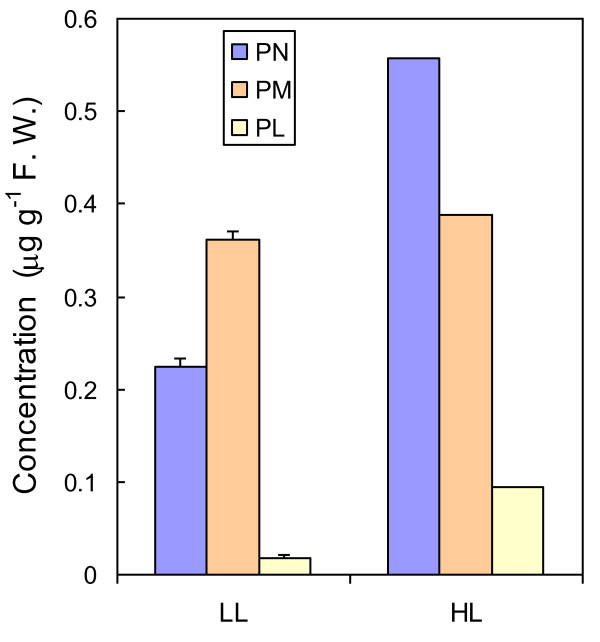
**Vitamin B6 components (expressed in μg/g fresh weight) in leaves of *Arabidopsis *plants grown in low light (LL) or acclimated for 7 d to high light (HL, 1000 μmol photons m^-2 ^s^-1 ^at 10°C)**. F. W. = fresh weight. PM, pyridoxamine; PN, pyridoxine; PL, pyridoxal. Data are mean values of 2 or 3 measurements + SD.

The expression of the *PDX1 *and *PDX2 *genes is up-regulated by several stress conditions, including high light [11,18,52]. However, so far the vitamin B6 concentration in plant tissues has not been measured under those conditions. Using HPLC, we were able to measure the non-phosphorylated forms of vitamin B6. Figure 11 shows the effect of high light (1000 μmol photons m^-2 ^s^-1 ^at 10°C for 7 d) on the concentration of nonphosphorylated vitamin B6 components of *Arabidopsis *leaves. Pyridoxine and pyridoxamine were the major vitamin B6 constituents measured in leaves, with pyridoxal being present in low amounts only. Pyridoxine and pyridoxal noticeably increased in high light while pyridoxamine did not change, so that the total (non-phosphorylated) vitamin B6 level increased by about 70%.

## Discussion

### Vitamin B6 deficiency leads to ^1^O_2_-mediated photodamage

Vitamin B6-deficient *Arabidopsis *leaves were more sensitive to treatments with the ^1^O_2 _generator eosin than WT leaves, and exogenous application of vitamin B6 reduced ^1^O_2 _level and mitigated lipid peroxidation in leaf discs exposed to high light. The protective role of vitamin B6 observed *in vitro *was confirmed *in vivo *in *Arabidopsis *plants challenged with endogenous ^1^O_2 _production induced by high light stress. Exposure of *Arabidopsis *plants to high light led to a rise in ^1^O_2_concentration and an accumulation of oxidized lipids, which were higher in *pdx1 *than in WT. The increased level of lipid peroxidation in mutant leaves was attributable to a ^1^O_2 _mediated attack on lipids. Those results show that vitamin B6 has a function in the protection of plants against ^1^O_2 _toxicity and photooxidative stress. This confirms *in vivo *the antioxidant capacity of vitamin B6 previously inferred from *in vitro *studies [9-17]. The role of vitamin B6 in the response of plants to light stress was further supported by our observation that the concentration of this vitamin is increased in *Arabidopsis *leaves exposed to high light intensity. This finding is in line with previous studies that have shown an increased expression of genes of the vitamin B6 biosynthesis pathway (*PDX1 *and *PDX2*) by abiotic stresses [11,18,52]. Illumination of *pdx1 *seedlings grown under sterile conditions has been reported to provoke degradation of the D1 protein of the PSII reaction center and to exacerbate the associated photoinhibition of PSII [18]. The latter phenomenon is attributed to ^1^O_2 _attack on the D1 protein itself, triggering structural changes in the PSII centre that initiate proteolytic degradation of the protein [53]. These data add further support to our conclusions that reduced levels of vitamin B6 in *pdx1 *leads to enhanced accumulation of ^1^O_2_.

### Direct versus indirect effect of vitamin B6 in photoprotection

The photoprotective role of vitamin B6 could be direct or indirect. A direct role would mean that vitamin B6 quenches ^1^O_2 _produced by light in the chloroplasts. This is plausible because this vitamin is able to quench ^1^O_2 _*in vitro *with a rather high efficiency [10]. The ^1^O_2_quenching rate constant of vitamin B6 is comparable to that of ascorbate and tocopherol [9]. However, because of the high reactivity of ^1^O_2_, this supposes that vitamin B6 is present *in planta *in the vicinity of the ^1^O_2 _production sites, namely the PSII reaction center and the chlorophyll antenna system in the chloroplasts [53]. Vitamin B6 levels in *Arabidopsis *leaves are relatively high ([20], this study), in the same range of concentrations as glutathione [48], but its sub-cellular distribution is unknown. To check if chloroplasts constitute a site of vitamin B6 accumulation in plant leaves, we prepared intact chloroplasts and we titrated vitamin B6 by HPLC (Additional File 4). Because our HPLC method requires large amounts of material (> 10 g of fresh weight), it was difficult to prepare sufficient amounts of intact chloroplasts from *Arabidopsis *leaves, and consequently we measured vitamin B6 in another plant species, tobacco, that is more suitable for purifying intact chloroplasts by ultracentrifugation on Percoll gradient. Both pyridoxine and pyridoxamine were detected in intact tobacco chloroplasts (Additional File 4). When normalized to the Chl content, the (nonphosphorylated) vitamin B6 content of chloroplasts (~0.16 μg/mg Chl) was approximately 3 times lower than the concentration in leaves. Considering that the chloroplast volume represents about 25% of the total cellular volume [54] and that Chl is localized exclusively in the chloroplasts, this suggests that there is a uniform distribution of vitamin B6 between the chloroplast and the rest of the cell. However, one cannot exclude that the level of vitamin B6 in chloroplasts was underestimated due to vitamin export during the chloroplast isolation. The occurrence of vitamin B6 in chloroplasts, as reported here, is consistent with a number of previous observations. First, the N-terminal amino acids of one of the enzymes of the vitamin B6 pathway, pyridoxine (pyridoxamine) 5'-phosphate oxidase, have been identified as a chloroplast transit peptide [55], suggesting a chloroplastic localization for this protein. Both components of the pyridoxal synthase complex, PDX1 and PDX2, have been shown to be attached to membranes, including chloroplastic membranes [11,21]. Furthermore, the present study has shown that vitamin B6 deficiency impacts the activity of Chl synthase, a plastid-localized protein. Since vitamin B6 is an efficient quencher of ^1^O_2 _*in vitro*, it is easy to speculate that the presence of a vitamin B6 pool in the chloroplast would reduce ^1^O_2 _levels. However, under conditions of severe light stress, ^1^O_2 _has been reported to leave thylakoid membranes and to migrate to the cytoplasm [56]. Therefore, since the light stress conditions used in this work to induce photooxidative damage were rather drastic (1500 μmol photons m^-2 ^s^-1 ^at 6°C), a leakage of ^1^O_2 _from the chloroplast to the cytosol cannot be excluded and therefore an action of vitamin B6 within the cytosol is also possible.

Hydroperoxides and endoperoxides generated in lipid peroxidation are known to undergo fragmentation to produce a broad range of reactive intermediates called reactive electrophile species [57,58]. Reactive electrophiles are harmful to macromolecules by reacting with nucleophilic groups, resulting in a variety of adducts and irreversible modifications. Compared to ROS, reactive electrophile species are stable and, due to their non-charged structure, some of them can migrate through hydrophobic membranes and hydrophilic media, so that they are able to propagate oxidative stress far from their site of formation [59]. Interestingly, pyridoxamine has been shown to trap lipid-derived carbonyl intermediates *in vitro *[60,61], and pyridoxamine adducts to lipid peroxidation products have been detected in the urine of pyridoxamine--treated animals [60]. In humans, pyridoxamine and pyridoxine are considered to be promising drug candidates for treatment of chronic conditions in which carbonyl compounds confer pathogenecity, such as diabetes [62,63]. A similar function as scavenger of intermediates in lipid peroxidation could be envisaged for vitamin B6 in plant cells. However, this mechanism does not explain the selective sensitivity of leaf discs to ^1^O_2 _(Fig. 4 vs. Additional File 2) since free radical-induced lipid peroxidation also generates reactive carbonyl species. Moreover, we administrated 4-hydroxynonenal, one of the most toxic carbonyl compounds produced from lipid peroxides [58], to detached *Arabidopsis *leaves, using the procedure described by Mano et al. [64]. As expected, necrosis developed concentrically from the application site of the hydroxynonenal solution on the leaf, but the extent of necrosis was similar in WT leaves and leaves of the *pdx1 *mutant (data not shown). Thus, vitamin B6 deficiency does not seem to enhance the sensitivity to reactive carbonyls, and an indirect function of vitamin B6 as scavenger of oxidized lipid derivatives seems unlikely.

One can also exclude the possibility that the increased level of ^1^O_2 _in leaves of the *pdx1 *mutant relative to WT leaves after illumination was due to an increased production of ^1^O_2_by the photosystems rather than a decreased quenching activity. In plants, ^1^O_2 _is produced mainly from chlorophyll triplet states, which are formed when the balance between light absorption by the photosystems and light utilization by the photosynthetic processes is upset in favor of the former process. This can be excluded in leaves of the *pdx1 *mutant since photosynthetic electron transport was not affected significantly relative to WT. Moreover, the total Chl concentration in *pdx1 *was lowered by ca. 20%, at least in young leaves, and this would be expected to reduce ^1^O_2 _production [65,66]. ^1^O_2 _can also be produced by Chl precursors such as Pchlide, as it is the case in the *flu Arabidopsis *mutant [67]. Based on our analyses of Chl biosynthesis intermediates, we can exclude this phenomenon in *pdx1*. The fact that exogenously applied ^1^O_2 _was more toxic to *pdx1 *than to WT is another indication that a change in ^1^O_2 _production by the photosystems cannot be the sole factor involved in the increased sensibility to ^1^O_2 _damage in *pdx1*. In this context, it is important to mention a recent work of Lytovchenko et al. [68] who showed that the profile of lipophilic compounds was not substantially affected in shoots of vitamin B6-deficient *Arabidopsis *plants. Therefore, we consider that the management of ^1^O_2_was less efficient in *Arabidopsis *leaves when vitamin B6 concentration was abnormally low.

The most efficient biological quenchers of ^1^O_2 _are thought to be the carotenoids and the vitamins C and E. Neither vitamin C (ascorbate) nor vitamin E (tocopherol) levels were reduced in *pdx1*. Although the total carotenoid content (on a leaf area basis) was lowered, the carotenoid concentration normalized to the Chl content was enhanced in *pdx1*. Among carotenoids, the xanthophyll zeaxanthin is known to play a crucial role in photoprotection [28,29,36,69]. Zeaxanthin synthesis and the associated NPQ were found to be stimulated in *pdx1*, and during long-term exposure to high light, the steady-state level of zeaxanthin was higher in *pdx1 *than in WT. Thus, the major antioxidant mechanisms involved in ^1^O_2 _elimination in leaves did not appear to be reduced in *pdx1*, supporting the notion that the reduced capacity of ^1^O_2 _quenching was directly related to the low concentration of vitamin B6, rather than to a secondary effect of vitamin B6 deficiency on the level of other antioxidant mechanisms. In sterile growth conditions, roots of *Arabidopsis *seedlings deficient in vitamin B6 displayed significant changes in lipid constituent content, such as a strong increase in α-tocopherol, supporting the idea that oxidative stress is involved in the inhibition of root growth [68].

### Vitamin B6 deficiency induces chronic light stress in leaves

Acclimation of WT *Arabidopsis *to high light induced marked changes in the protein composition of thylakoids. As previously reported [e.g. [70,71]], the most obvious modification was a decrease in the PSII antenna size, leading to a higher Chl *a*/Chl *b *ratio. The abundance of all Lhcb proteins, except CP26 and to a lesser extent CP29, was decreased in high light. CP26 is supposed to constitute with CP29 an inner part of the antenna system that undergoes limited modifications with environmental conditions [71]. Interestingly, the loss of PSII antennae was observed in low light when thylakoids prepared from leaves of *pdx1 *were compared with WT thylakoids and was strongly exacerbated when *pdx1 *was exposed to high light (1000 μmol m^-2 ^s^-1^). The gradient profile and characteristics of the photosynthetic complexes from low-light-grown *pdx1 *were very similar to that of high-light-acclimated WT thylakoids. Consistent with these observations the decreased Chl levels in *pdx1 *versus WT was strongly dependent on light intensity: in very low light (~100 μmol photons m^-2 ^s^-1^), WT leaves and mutant leaves had very similar Chl *a*/*b *ratio and total chlorophyll content whereas the Chl *a*/*b *value differed drastically in high light. Thus, comparison of the photosynthetic complexes between *pdx1 *and WT suggests that, for a given PFD, the mutant senses a higher level of light stress than WT. Since the ^1^O_2 _level induced by light in *pdx1 *was enhanced relative to WT, it is possible that the loss of Chl antennae represents a response to ^1^O_2 _stress in the mutant. Although long-term acclimation of vascular plants to ^1^O_2_has not yet been investigated, ^1^O_2 _is known to induce changes in gene expression. Particularly, the gene coding for the PSII antenna Lhcb2 has been shown to be strongly and specifically downregulated by ^1^O_2_[67]. In the green alga *Chlamydomonas*, the early phases of ^1^O_2_-mediated photooxidative stress were associated with the repression of the Lhcbm1 and Lhcbm2 genes at the RNA level [72]. UV-B radiation, which is know to induce the production of ROS including ^1^O_2_, has been shown to downregulate expression of several Lhcb genes [52]. Interestingly, these conditions also up-regulated the expression of a *PDX1 *homologue, *PYROA *[52]. Alternatively, the loss of PSII antennae could also result from the inhibition of Chl synthesis in the *pdx1 *mutant. However, previous work on different transgenic plants have shown that a decreased availability of Chl induces a decrease in the amount of photosynthetic complexes embedded in the thylakoid membranes, but it does not change the PSII antenna size [73,74].

## Conclusion

The potential function of the vitamin B6 constituents as antioxidants has been reported in several *in vitro *studies in which yeast or animal cells were treated with different ROS [12-16]. There are also a few preliminary studies performed *in vitro*, that support the idea that vitamin B6 could fulfill a similar role in plant cells [11,17,21]. The present study of whole *Arabidopsis *plants provides the first evidences for an active and specific antioxidant role of vitamin B6 *in planta*. Vitamin B6 deficiency was associated with a marked decrease in the tolerance to photooxidative stress, which manifested itself as an increase in the ^1^O_2 _level in high light and a marked enhancement in ^1^O_2_-mediated lipid peroxidation. On the other hand, it is known that there are some redundancies between the antioxidant systems in chloroplasts, so that removing one antioxidant mechanism is generally compensated, at least partially, by an increase in other protections. This has been established in *Arabidopsis *and cyanobacteria for two classes of ^1^O_2 _quenchers, the carotenoids and the tocopherols [47,75]. Similarly, removal of vitamin B6 from an *Arabidopsis *mutant deficient in both carotenoids and tocopherols resulted in an extreme sensitivity to high light stress. These result indicate that vitamin B6 may play a specific role in antioxidant defense that is not completey fulfilled by carotenoids or tocopherols. Consequently, vitamin B6 can be considered as a new member of the network of protective compounds involved in the management of ^1^O_2 _in plants.

## Methods

### Plant material, growth conditions and treatments

Wild-type *Arabidopsis thaliana *(ecotype Col-0) and the *pdx1.3 *(At5g01410) T-DNA line were grown in a phytotron under controlled conditions: PFD was 150-200 μmol photons m^-2 ^s^-1^, photoperiod 8 h, air temperature 23/28°C (day/night) and relative air humidity 75%. Most of the experiments were performed on plants aged 5 weeks. Light stress was imposed by transferring plants to a growth chamber at 6/12°C (day/night) under a PFD of 1500 μmol photons m^-2 ^s^-1 ^and a photoperiod of 8 h. In preliminary experiments where we checked a number of light/temperature conditions, we selected this stress condition that appeared to be the most suitable to discriminate between WT and *pdx1 *in terms of photosensitivity. The *pdx1 *mutant was crossed with the *vte1 npq1 *double mutant (see [47]) to generate the triple mutant *vte1 npq1 pdx1 *deficient in vitamin E, zeaxanthin and vitamin B6. The triple mutant and the double/single mutants were exposed to light stress by transferring them to a PFD of 1000 μmol photons m^-2 ^s^-1 ^at 10°C.

Leaf discs of 1 cm in a diameter were treated with a solution of 3.5% H_2_O_2_, 50 μM methylviologen or 0.5% eosin Y, as previously described [31]. The infiltrated discs were exposed to white light of PFD 400 μmol photons m^-2 ^s^-1 ^(for the eosin or methylviologen treatment) or 100 μmol m^-2 ^s^-1 ^(for the H_2_O_2 _treatment). Attached leaves were slowly infiltrated with 100 μM SOSG (Singlet Oxygen Sensor Green, Invitrogen) and/or vitamin B6 (1 mM pyridoxal) under pressure with a syringe. A 1-ml syringe, without needle and filled with the solution to be infiltrated, was pushed against the lower surface of the leaf, and the solution (200 μl) was forced to enter inside of the leaf under pressure. Plants with SOSG-infiltrated leaves were kept in darkness for 1 h and then exposed for 40 min to white light of PFD 450 μmol photons m^-2 ^s^-1^. For high light treatment of leaf discs, the discs (diameter, 1 cm) were exposed at constant temperature (10°C) to white light (PFD, 1000 μmol photons m^-2 ^s^-1^), as previously described [47]. In some cases, leaf discs were preinfiltrated with 2 mM vitamin B6 (pyridoxal) for 1 h. PFDs were measured with a Li-Cor quantum meter (Li-185B/Li-190SB).

### Chlorophylls, carotenoids and vitamin E

One leaf disc (diameter, 1 cm) was ground in 400 μl of cold methanol. After filtration through a 0.45-μm PTFE filter (Iso-Disc, SUPELCO), 80 μl of the extract was immediately analyzed by HPLC, as previously described [47]. Pigments were detected at 445 nm and α-tocopherol was detected by fluorescence (λ_ex _= 295 nm, λ_em _= 340 nm). Running time was 22 min, flow rate was 1.5 ml.min^-1^.

### Chlorophyll precursors

Chlorophyll esters and (proto)chlorophyllide were quantitated using reverse phase HPLC analysis according to [76], except that detection was performed by absorbance at 430 nm.

### Ascorbic Acid

Ascorbate was analyzed by HPLC as described elsewhere [47]. Total ascorbate was measured by reducing dehydroascorbic acid to ascorbic acid with TCEP (Tris-carboxyethylphosphine). Three leaf discs of 1 cm in diameter (about 100 mg) were ground in 750 μL of 0.1 M metaphosphoric acid. Samples were filtered through a 0.2 μm nylon membrane (Spin-X Costar). A 6 μL sample was immediately injected, and 6 μL were treated for 4 h with 10 mM TCEP in darkness at 25°C. Ascorbate was detected at 245 nm in sulphuric acid-acidified water (pH 2.5) with a retention time of 5 min under a flow of 0.65 mL min^-1^.

### Lipid peroxidation analyses

Lipids were extracted from 0.5 g frozen leaves by grinding with 2 × 1 mL chloroform containing 1 mg/mL triphenyl phosphine and 0.05% (w/v) butylated hydroxytoluene, with 15-hydroxy-11,13(Z, E)-eicosadienoic acid as internal standard. The organic phase was evaporated under a stream of N_2_. The residue was recovered in 1.25 mL ethanol and 1.25 mL 3.5 M NaOH and hydrolyzed at 80°C for 15 min. After addition of 2.2 mL 1 M citric acid, hydroxyl fatty acids were extracted with 2 × 1 mL hexane/ether (50/50). An aliquot of the organic phase (50 μl) was submitted to straight phase HPLC (Waters, Millipore, St Quentin-Yvelines, France) using a Zorbax rx-SIL column (4.6·250 mm, 5 μm particle size, Hewlett Packard, Les Ullis, France), isocratic elution with 70/30/0.25 (v/v/v) hexane/diethyl ether/acetic acid at a flow rate of 1.5 ml min^-1^, and UV detection at 234 nm. ROS-induced lipid peroxidation was evaluated from the levels of the different hydroxyoctadecatrienoic acid (HOTE) isomers as previously described using 15-hydroxy-11,13(Z, E) eicosadienoic acid as internal standard [77]. LOX-induced lipid peroxidation was estimated from the level of 13-HOTE after substraction of racemic 13-HOTE (attributable to ROS-mediated lipid peroxidation), as explained in [77].

The distribution of hydroxy fatty acid isomers was analyzed by HPLC-electrospray ionization-MS/MS as detailed previously [42]. Aliquots from the hydroxyl fatty acid extracts were evaporated and recovered in aqueous 1 mM ammonium acetate/acetronitrile (60/40, v/v) with [^18^O_2_]13-HOTE used as internal standard. Hydroxy fatty acids were separated by HPLC and analyzed using a Waters Micromass Quatro premier triple quatrupole mass spectrometer in the negative electrospray ionization mode.

### Thermoluminescence and autoluminescence imaging

Lipid peroxidation was measured in leaf discs by thermoluminescence using a custom-made apparatus that has been described previously [40]. The amplitude of the thermoluminescence band peaking at ca. 135°C was used as an index of lipid peroxidation [40,78]. The samples (2 leaf discs of 8 mm in diameter) were slowly heated from 25°C to 150°C at a rate of 6°C min^-1^. Photon emission associated with lipid peroxidation was also imaged at room temperature using a highly sensitive charge coupled device (CCD) camera (VersArray LN/CCD 1340-1300B, Roper Scientific), with a liquid N_2 _cooled sensor to enable measurement of faint light by signal integration [34]. Treated plants were dark-adapted for 2 h before imaging, to allow chlorophyll luminescence to fade away. Acquisition time was 20 min. Full resolution of the CCD is 1300 × 1340 pixels. On-CCD binning of 2 × 2 pixels was used to increase detection sensitivity, so that the resulting resolution was 650 × 670 pixels.

### Photosynthetic electron transport

Chl fluorescence from attached leaves was measured in air at room temperature with a PAM-2000 fluorometer (Walz) [47]. The quantum yield of PSII photochemistry was calculated in white light as ΔF/Fm', where ΔF is the difference (Fm'-Fs) between the maximal fluorescence level Fm' (measured with a 800-ms pulse of saturating light) and Fs, the steady-state fluorescence level. White light was produced by a Schott KL1500 light source. NPQ was calculated as (Fm/Fm')-1 where Fm is the maximal fluorescence level in the dark [47].

O_2 _exchange by leaf discs was measured in a Clark-type O_2 _electrode (Hansatech LD2/2) under CO_2 _saturating conditions. CO_2 _was generated in the cell with a carbonate/bicarbonate buffer. White light was produced by a Hansatech LS2 light source combined with neutral density filters.

### Membrane preparation and solubilisation

*Arabidopsis *leaves were shortly grinded in a solution containing 20 mM Tricine KOH pH 7.8, 0.4 M NaCl, 2 mM MgCl_2 _and the protease inhibitors 0.2 mM benzamidine, 1 mM є-aminocaproic acid. The solution was filtered through miracloth tissue and centrifuged 10 min at 1400 g. The pellet was resuspended in a solution containing 20 mM Tricine KOH pH 7.8, 0.15 M NaCl, 5 mM MgCl_2 _and protease inhibitors as before and then centrifuged 10 min at 4000 g. The pellet was resuspended in 20 mM Hepes 7.5, 15 mM NaCl, 5 mM MgCl_2 _and centrifuged again 10 min at 6000 g and stocked in 20 mM Hepes 7.5, 0.4 M Sorbitol, 15 mM NaCl, 5 mM MgCl_2_.

Membranes corresponding to 150 μg Chls were washed once with 5 mM EDTA, 10 mM Hepes pH 7.5, resuspended at 1 mg/ml Chls in 10 mM Hepes pH 7.5 and then solubilized at 0.5 mg/ml Chls by adding an equal volume of dodecyl-α-D-maltoside solution to have at a final detergent concentration of 0.8% or 1.2% and vortexing for a few seconds. The solubilised samples were centrifuged at 15.000 × g for 10 min to eliminate unsolubilised material and then fractionated by ultracentrifugation in a sucrose gradient (20 h, 288.000 × g, 4°C). The gradient was formed directly in the tube by freezing at -80°C and thawing at 4°C a 0.5 M sucrose solution containing 0.06% α-DM and 10 mM Hepes pH 7.5.

Chlorophylls and carotenoids were extracted in acetone (80% final concentration buffered with Na_2_CO_3_) and measured by fitting of the absorption spectrum of acetone extracts [79].

### SDS-Page

Electrophoresis were performed using the Tris-Tricine system at 14% acrylamide concentration [80] or the Laemmli system [81] with the modification as in [82].

### Vitamin B6

HPLC measurements of nonphosphorylated vitamin B6 components were carried out on leaves or isolated chloroplasts as described elsewhere [19,20]. Vitamin B6 was extracted from approximately 10 g of leaves (fresh weight). Intact chloroplasts were prepared from about 100 g of tobacco leaves, as described previously [83].

## Abbreviations

Chl: chlorophyll; Lhcb: Light harvesting complex of PSII; PS: photosystem; PFD: photon flux density; ^1^O_2_: singlet oxygen; WT: wild type; PChlide and Chlide: protochlorophyllide and chlorophyllide; ROS: reactive oxygen species; HOTE: hydroxy octadecatrienoic acid; SOSG: singlet oxygen sensor green; NPQ: nonphotochemical quenching.

## Authors' contributions

MH designed and performed the experiments. BK and CT performed HPLC analyses of hydroxy fatty acids. AS and DR measured vitamin B6 concentration in leaves and chloroplasts. FF analyzed Chl precursors. SC performed the characterization of the photosynthetic complexes. MH wrote the manuscript. All authors read and approved the final version of the manuscript.

## Supplementary Material

Additional file 1 **Effects of the *pdx1 *mutation on growth of *Arabidopsis *plants on soil**. A) Shoot growth as measured by the rosette diameter (in cm), B) Roots after 4-week growth. Root length and dry weight are expressed in cm and mg dry weight (D.W.) per plant, respectively. Click here for file

Additional file 2 **Oxidative stress in *Arabidopsis *leaf discs (WT and *pdx1*) exposed to hydrogen peroxide (3.5%) or to the superoxide-generating methylviologen herbicide (50 μM)**. A) Autoluminescence imaging of leaf discs exposed for 0, 5, 24 and 66 h to hydrogen peroxide in low light (100 μmol m^-2 ^s^-1^), B) Autoluminescence intensity of leaf discs exposed for 0 or 24 h to hydrogen peroxide in low light (data are mean values of 10 measurements + SD), C) Autoluminescence imaging of leaf discs exposed to methylviologen in the light (PFD, 400 μmol photons m^-2 ^s^-1^) for 0, 2.5, 5 and 7 h. D) Autoluminescence intensity of leaf discs exposed for 5 h to methylviologen in the light. Data are mean values of 10 measurements + SD. Click here for file

Additional file 3 **Absorption spectrum of the pigments extracted from A) the B2 band, B) the B3 band and C) the B6 band of the sucrose gradients **(see Fig. 10A). Pigments were extracted in acetone as explained elsewhere [79]. Click here for file

Additional file 4 **Nonphosphorylated vitamin B6 concentration (normalized to the Chl content) in tobacco leaves and in intact chloroplasts prepared from tobacco leaves**. PN = Pyridoxine; PM = Pyridoxamine. Click here for file
