# Supplementary material for: Vitamin B6 deficient plants display increased sensitivity to high light and photo-oxidative stress
Source: BMC Plant Biol. 2009 Nov 10;9:130. doi: 10.1186/1471-2229-9-130 (PMC2777905; doi:10.1186/1471-2229-9-130)
Supplement: Additional file 1 — Effects of the pdx1 mutation on growth of Arabidopsis plants on soil. A) Shoot growth as measured by the rosette diameter (in cm), B) Roots after 4-week growth. Root length and dry weight are expressed in cm and mg dry weight (D.W.) per plant, respectively. [file 1471-2229-9-130-S1.ppt]

## Slide 1
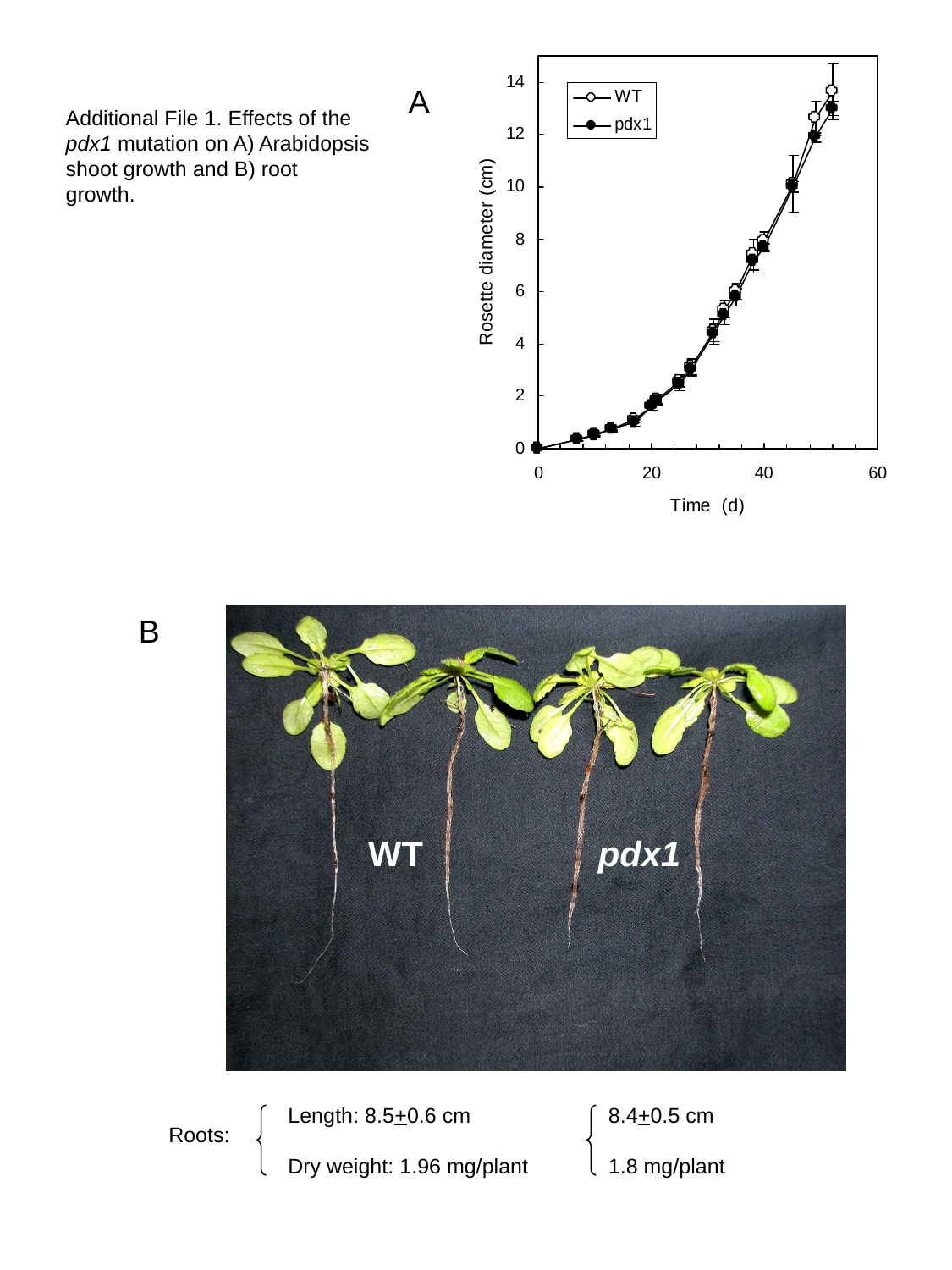

A
Additional File 1. Effects of the pdx1 mutation on A) Arabidopsis shoot growth and B) root growth.
B
WT
pdx1
Length: 8.5+0.6 cm
Dry weight: 1.96 mg/plant
8.4+0.5 cm
1.8 mg/plant
Roots:
