# Supplementary figures and images for: Vitamin B6 deficient plants display increased sensitivity to high light and photo-oxidative stress
Source: BMC Plant Biol. 2009 Nov 10;9:130. doi: 10.1186/1471-2229-9-130 (PMC2777905; doi:10.1186/1471-2229-9-130)

## Slide 1
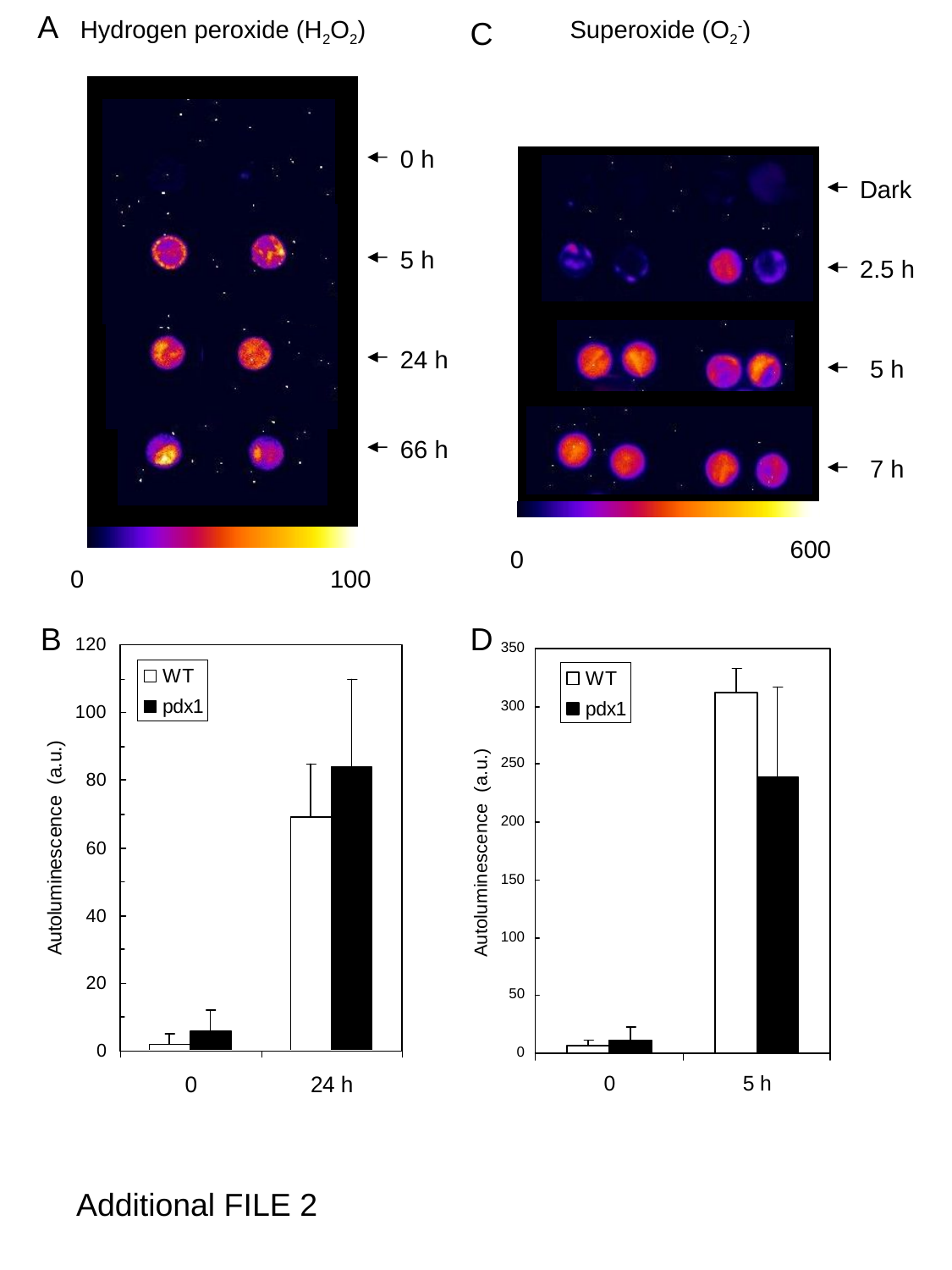

A
Hydrogen peroxide (H2O2)
C
Superoxide (O2-)
0 h
Dark
5 h
2.5 h
24 h
5 h
66 h
7 h
600
0
0
100
B
D
Additional FILE 2

Supplement: Additional file 2 — Oxidative stress in Arabidopsis leaf discs (WT and pdx1) exposed to hydrogen peroxide (3.5%) or to the superoxide-generating methylviologen herbicide (50 μM). A) Autoluminescence imaging of leaf discs exposed for 0, 5, 24 and 66 h to hydrogen peroxide in low light (100 μmol m-2 s-1), B) Autoluminescence intensity of leaf discs exposed for 0 or 24 h to hydrogen peroxide in low light (data are mean values of 10 measurements + SD), C) Autoluminescence imaging of leaf discs exposed to methylviologen in the light (PFD, 400 μmol photons m-2 s-1) for 0, 2.5, 5 and 7 h. D) Autoluminescence intensity of leaf discs exposed for 5 h to methylviologen in the light. Data are mean values of 10 measurements + SD. [file 1471-2229-9-130-S2.ppt]
