# Supplementary material for: Vitamin B6 deficient plants display increased sensitivity to high light and photo-oxidative stress
Source: BMC Plant Biol. 2009 Nov 10;9:130. doi: 10.1186/1471-2229-9-130 (PMC2777905; doi:10.1186/1471-2229-9-130)
Supplement: Additional file 3 — Absorption spectrum of the pigments extracted from A) the B2 band, B) the B3 band and C) the B6 band of the sucrose gradients (see Fig. 10A). Pigments were extracted in acetone as explained elsewhere [79]. [file 1471-2229-9-130-S3.doc]

*Additional File 3: absorption spectra of B2, B3 and B6 bands of the sucrose gradients (see Fig. 10A). Abs = absorbance. Ctr= control conditions; hl = high light stress conditions.*

B2 band : Lhc monomeric.

B3 band: LHCII.

B6 band: PSI-LHCI.
