# Supplementary material for: Vitamin B6 deficient plants display increased sensitivity to high light and photo-oxidative stress
Source: BMC Plant Biol. 2009 Nov 10;9:130. doi: 10.1186/1471-2229-9-130 (PMC2777905; doi:10.1186/1471-2229-9-130)
Supplement: Additional file 4 — Nonphosphorylated vitamin B6 concentration (normalized to the Chl content) in tobacco leaves and in intact chloroplasts prepared from tobacco leaves. PN = Pyridoxine; PM = Pyridoxamine. [file 1471-2229-9-130-S4.ppt]

## Slide 1
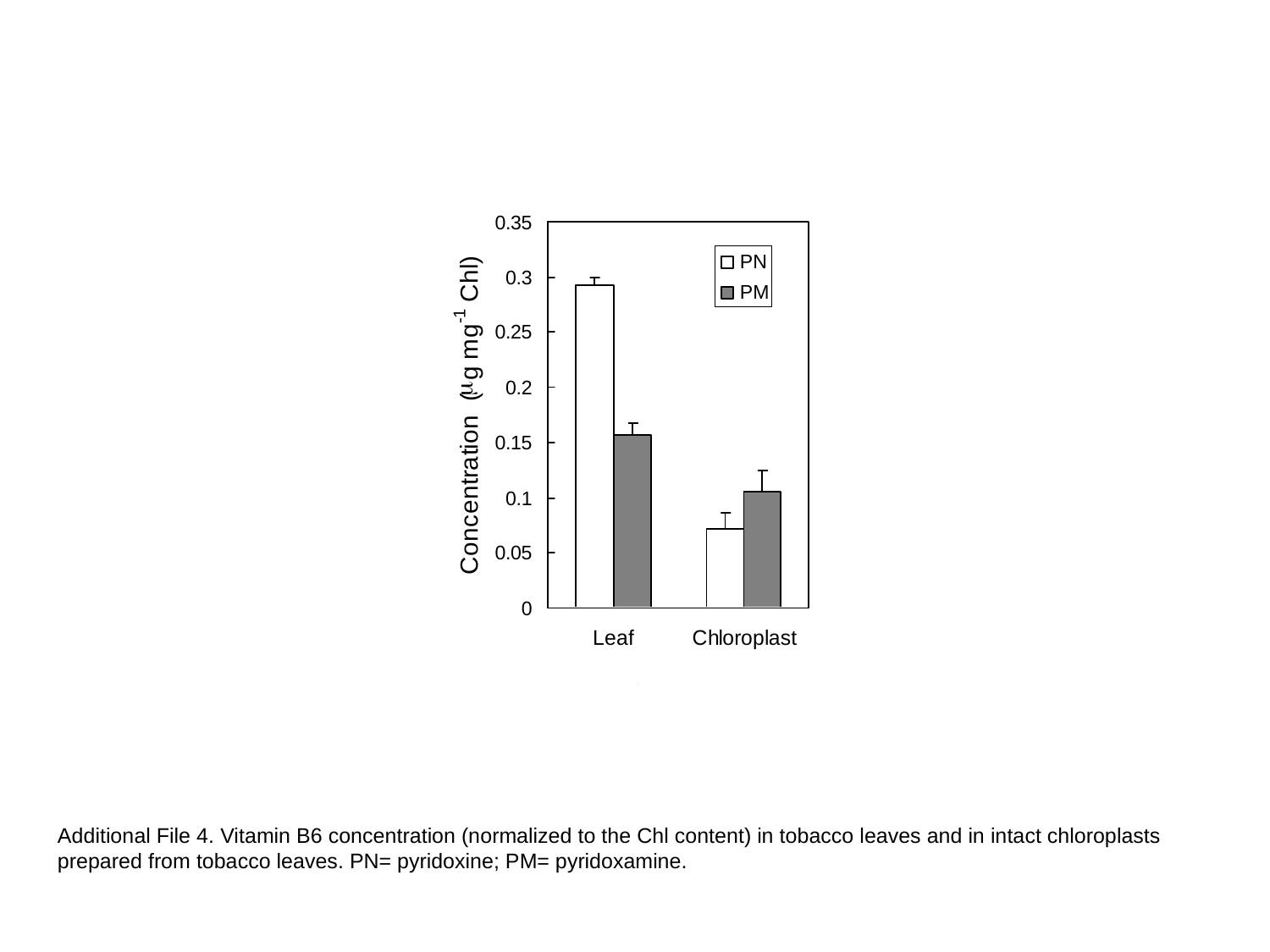

Additional File 4. Vitamin B6 concentration (normalized to the Chl content) in tobacco leaves and in intact chloroplasts prepared from tobacco leaves. PN= pyridoxine; PM= pyridoxamine.
